# Acute Catarrh of the Stomach

**Published:** 1890-03-22

**Authors:** 


### DIETETICS.

### ACUTE CATARRH OF THE STOMACH.

Dr. Robert Saundby, of Birmingham, recommends in that common ailment, acute catarrh of the stomach, a diet restricted to milk and lime water, or, better still, peptonised milk. If, for any reason, this cannot be adhered to, the following may be allowed: *Breakfast.*—A cup of cocoa or bread and milk, both to be taken cold; a little boiled fish or lightly poached egg, with small slice of toast. *Luncheon.*—A cupful of Benger's peptonised food or a custard pudding. *Dinner.*—Boiled fowl or stewed sweetbread with toast. Milk pudding. No beer, wine, or spirits. Aërated waters. All fluids in small quantities. In addition, order the following powders:—

Rg. Hydrarg. subchlor........................ gr. ij.

Sacch. alb........................................ gr. v.

F. pulv........................mitte ij.

Sign.—One to be taken at bed time.

Rg. Bismuthi carb. ............................. gr. x.

Sodii. bicarb...................................... gr. x.

Pulv. rhei........................................ gr. iij.

Pulv. cinnam. co. ............................. gr. v.

If there be much pain, apply a mustard leaf over the pit of the stomach.

